# Sleeping with one eye open: loneliness and sleep quality in young adults

**DOI:** 10.1017/S0033291717000629

**Published:** 2017-05-17

**Authors:** T. Matthews, A. Danese, A. M. Gregory, A. Caspi, T. E. Moffitt, L. Arseneault

**Affiliations:** 1 MRC Social, Genetic and Developmental Psychiatry Centre, Institute of Psychiatry, Psychology and Neuroscience, King's College London, London, UK; 2 Department of Child and Adolescent Psychiatry, Institute of Psychiatry, Psychology and Neuroscience, King's College London, London, UK; 3 National and Specialist Child Traumatic Stress and Anxiety Clinic, South London and Maudsley NHS Foundation Trust, London, UK; 4 Department of Psychology, Goldsmiths University, London, UK; 5 Departments of Psychology and Neuroscience, Psychiatry and Behavioral Sciences, and Institute for Genome Sciences and Policy, Duke University, Durham, NC, USA

**Keywords:** Loneliness, sleep quality, mental health, violence victimization, young adulthood

## Abstract

**Background:**

Feelings of loneliness are common among young adults, and are hypothesized to impair the quality of sleep. In the present study, we tested associations between loneliness and sleep quality in a nationally representative sample of young adults. Further, based on the hypothesis that sleep problems in lonely individuals are driven by increased vigilance for threat, we tested whether past exposure to violence exacerbated this association.

**Method:**

Data were drawn from the Environmental Risk (E-Risk) Longitudinal Twin Study, a birth cohort of 2232 twins born in England and Wales in 1994 and 1995. We measured loneliness using items from the UCLA Loneliness Scale, and sleep quality using the Pittsburgh Sleep Quality Index. We controlled for covariates including social isolation, psychopathology, employment status and being a parent of an infant. We examined twin differences to control for unmeasured genetic and family environment factors.

**Results:**

Feelings of loneliness were associated with worse overall sleep quality. Loneliness was associated specifically with subjective sleep quality and daytime dysfunction. These associations were robust to controls for covariates. Among monozygotic twins, within-twin pair differences in loneliness were significantly associated with within-pair differences in sleep quality, indicating an association independent of unmeasured familial influences. The association between loneliness and sleep quality was exacerbated among individuals exposed to violence victimization in adolescence or maltreatment in childhood.

**Conclusions:**

Loneliness is robustly associated with poorer sleep quality in young people, underscoring the importance of early interventions to mitigate the long-term outcomes of loneliness. Special care should be directed towards individuals who have experienced victimization.

## Introduction

Loneliness is defined as a distressing feeling that is experienced when social connections are perceived to be inadequate (Peplau & Perlman, 1982). An evolutionary account of loneliness proposes that for members of a social species, being embedded within a group provides safety, and the perception of being socially cut off gives rise to feelings of vulnerability (Cacioppo *et al.* 2006*a*). This triggers changes in cognition and behaviour that serve to guard the individual against potential threats (Cacioppo & Hawkley, 2009). One hypothesis implied by this model is that feelings of loneliness are associated with impaired sleep quality: as sleep is a state in which it is impossible to remain vigilant, the unsafe feeling of loneliness is at odds with restful sleep. Research has shown that lonely individuals report poorer subjective sleep quality and experience more fragmented sleep, as measured via actigraphy, while their total duration of sleep is unaffected (Cacioppo *et al.* 2002; Pressman *et al.* 2005; Hawkley *et al.* 2010*a*; Kurina *et al.* 2011). Thus, while lonely individuals do not appear to sleep more or less than non-lonely individuals, their sleep may be less restful, consistent with the hypothesis that raised vigilance for threat intrudes on the sleep state.

In the present study, we investigated associations between loneliness and sleep quality in a nationally representative cohort of young adults. This age group is of particular interest for two reasons. First, loneliness is especially prevalent at this stage of life, concomitant with shifts in young adults’ social needs and environments (Office for National Statistics, 2014; Qualter *et al.* 2015). Second, a significant proportion of individuals experience loneliness persistently over time (Newall *et al.* 2013), and chronic social disconnection predicts poor health outcomes in a dose–response manner (Caspi *et al.* 2006; Danese *et al.* 2009). Therefore, individuals who become lonely early in life may be particularly at risk for ill health in the future. Prior studies which have found associations between loneliness and sleep in this age group have used opportunity samples of university undergraduates (Cacioppo *et al.* 2002; Pressman *et al.* 2005); however, it is important to ascertain whether the profile of sleep impairments generalizes across the full range of socio-economic and occupational circumstances in the young population.

Research has shown that the associations between loneliness and sleep problems are not accounted for by plausible confounders such as depression, body mass index or health-related behaviours (Cacioppo *et al.* 2002; Hawkley *et al.* 2010*a*; Kurina *et al.* 2011). However, sleep impairments are included among the diagnostic criteria for a number of mental health disorders, including generalized anxiety and post-traumatic stress disorder (American Psychiatric Association, 2013), underscoring the need to control comprehensively for symptoms of psychopathology in order to test the independence of the association between loneliness and sleep. Other sources of confounding are more difficult to control for, including genetic influences. There is evidence for substantial heritability of both loneliness (Goossens *et al.* 2015; Matthews *et al.* 2016) and sleep quality (Barclay *et al.* 2010), and their association may be partly explained by shared genetic aetiologies. Furthermore, unmeasured factors in the family environment, such as parental influences or life events, may also contribute jointly to the experiences of loneliness and sleep quality. One approach by which these confounds can be controlled is by comparing individuals from the same family, using a design such as monozygotic (MZ) twin differences (Vitaro *et al.* 2009). As members of an MZ twin pair share identical genomes and grow up in the same family home, any differences within pairs is attributable to experiences unique to individuals. Measuring MZ twin differences on two traits allows their association to be tested while holding family-wide influences constant.

Not all lonely individuals necessarily experience sleep problems, and it is possible that other factors play a role in exacerbating their susceptibility to sleep impairments. Given that one of the posited reasons for the restless sleep of lonely individuals is a perception of threat in the environment, past exposure to actual threats may intensify this perception and further compromise the restfulness of sleep. Exposure to violence victimization is one plausible candidate. Imaging studies have shown that childhood maltreatment is associated with increased amygdala activation in response to threatening social stimuli, suggesting heightened vigilance for threat (McCrory *et al.* 2011). This lends itself to the possibility that perceptions of threats triggered by loneliness may be particularly pronounced among individuals with a history of violence victimization, magnifying the disruption of sleep. Using longitudinal data, we tested for an exacerbating influence of violence victimization on the relationship between loneliness and sleep. Specifically, we examined recent victimization experiences in adolescence, and tested whether the effect could be extended earlier in life with childhood maltreatment.

## Method

### Participants

Participants were members of the Environmental Risk (E-Risk) Longitudinal Twin Study, which tracks the development of a birth cohort of 2232 British children. The sample was drawn from a larger birth register of twins born in England and Wales in 1994–1995 (Trouton *et al.* 2002). Full details about the sample have been reported elsewhere (Moffitt & E-Risk Study Team, 2002). Briefly, the E-Risk sample was constructed in 1999–2000, when 1116 families (93% of those eligible) with same-sex 5-year-old twins participated in home-visit assessments. This sample comprised 56% MZ and 44% dizygotic (DZ) twin pairs; sex was evenly distributed within zygosity (49% male).

Families were recruited to represent the UK population with newborns in the 1990s, to ensure adequate numbers of children in disadvantaged homes and to avoid an excess of twins born to well-educated women using assisted reproduction. The study sample represents the full range of socio-economic conditions in Great Britain, as reflected in the families’ distribution on a neighbourhood-level socio-economic index [ACORN (A Classification of Residential Neighbourhoods), developed by CACI Inc. for commercial use] (Odgers *et al.* 2012*a*, *b*). Specifically, E-Risk families’ ACORN distribution matches that of households nationwide: 25.6% of E-Risk families live in ‘wealthy achiever’ neighbourhoods compared with 25.3% nationwide; 5.3% *v*. 11.6% live in ‘urban prosperity’ neighbourhoods; 29.6% *v*. 26.9% live in ‘comfortably off’ neighbourhoods; 13.4% *v*. 13.9% live in ‘moderate means’ neighbourhoods, and 26.1% *v*. 20.7% live in ‘hard-pressed’ neighbourhoods. E-Risk under-represents ‘urban prosperity’ neighbourhoods because such houses are likely to be childless.

Follow-up home visits were conducted when the children were aged 7 years (98% participation), 10 years (96% participation), 12 years (96% participation), and, most recently in 2012–2014, at 18 years (93% participation). There were 2066 participants who took part in the E-Risk assessments at age 18 years, and the proportions of MZ (55%) and male same-sex (47%) twins were almost identical to those found in the original sample at age 5 years. The average age of the twins at the time of the assessment was 18.4 years (s.d. = 0.36); all interviews were conducted after their 18th birthday. There were no differences between those who did and did not take part at age 18 years in terms of socio-economic status assessed when the cohort was initially defined (χ^2^ = 0.86, *p* = 0.65), intelligence quotient scores at age 5 years (*t* = 0.98, *p* = 0.33), and emotional or behavioural problems at age 5 years (*t* = 0.40, *p* = 0.69 and *t* = 0.41, *p* = 0.68, respectively). Home visits at ages 5, 7, 10 and 12 years included assessments with participants as well as their mother (or primary caretaker); the home visit at age 18 years included interviews only with the participants. Each twin was assessed by a different interviewer.

The Joint South London and Maudsley and the Institute of Psychiatry Research Ethics Committee approved each phase of the study. Parents gave informed consent and twins gave assent between 5 and 12 years and then informed consent at age 18 years.

### Measures

#### Loneliness

We measured current feelings of loneliness at age 18 years using four items from the UCLA Loneliness Scale, version 3 (Russell, 1996): ‘How often do you feel that you lack companionship?’, ‘How often do you feel left out?’, ‘How often do you feel isolated from others?’ and ‘How often do you feel alone?’. These items were rated ‘hardly ever’ (0), ‘some of the time’ (1) or ‘often’ (2). The items were administered as part of a self-completed computer-based questionnaire. We summed the responses to produce a total loneliness score (Cronbach *α* = 0.83).

We also assessed loneliness using interviewers’ reports. After the home visits, the study interviewers (*n* = 14) completed an inventory of questions about their overall impressions of the participants’ personality and behaviour, based on their observations during the structured interview. Interviewers were trained to familiarize themselves with the questions in order to know what to observe, and took comprehensive notes on which to base their responses. The questions were completed immediately after the home visit in order to maximize recall. The interviewers had not met the participants prior to the visit. We used three of the items to construct a measure of interviewer-rated loneliness: ‘seems lonely’, ‘feels that no one cares for them’ and ‘has trouble making friends’. Items were coded ‘no’ (0), ‘a little/somewhat’ (1) and ‘yes’ (2). As the self-report loneliness measure was administered via computer, interviewers were blind to participants’ responses. We summed the three items to create a total scale (Cronbach *α* = 0.70). The correlation between the self-report and interviewer ratings of loneliness was 0.46.

#### Sleep quality

We measured sleep quality at age 18 years using the Pittsburgh Sleep Quality Index (PSQI; Buysse *et al.* 1989). The PSQI consists of 18 self-report items relating to individuals’ sleep patterns and different forms of sleep impairment in the past month. These questions are used to derive scores for seven different components of sleep (subjective sleep quality, sleep latency, sleep duration, habitual sleep efficiency, sleep disturbances, use of sleep medication and daytime dysfunction), each scored from 0 to 3. These were summed to produce a global score ranging from 0 to 21, with higher scores reflecting worse sleep quality. The mean global PSQI score in the present sample was 5.39 (s.d. = 3.18).

#### Covariates

To test the independence of the association between loneliness and sleep quality at age 18 years, we controlled for social isolation, based on the hypothesis that the subjective experience of loneliness would be associated with sleep quality over and above individuals’ actual degree of social connection. We further controlled for symptoms of depression, anxiety, alcohol abuse and dependence, attention-deficit/hyperactivity disorder (ADHD) and post-traumatic stress disorder (PTSD). We also controlled for individuals who were not in employment, education or training (NEET) or who were a parent of an infant, two circumstances which could lead to changes in social activities and sleeping schedules. Full details of covariates are presented in Table 1.

**Table 1.** Descriptive statistics of covariates

	Measure	Range	Mean (s.d.) or %	Reference
Social isolation	Multidimensional Scale of Perceived Social Support. Items reverse-scored with higher scores reflecting greater isolation	0–24	3.29 (4.35)	Zimet *et al.* (1998)
Psychopathology	Diagnostic Interview Schedule for DSM-IV.	Depression: 0–9	1.81 (2.97)	American Psychiatric Association (1994); Robins *et al.* (1995)
	Depression, anxiety, alcohol use and ADHD are measured as symptom scales; PTSD is a diagnosis	Anxiety: 0–6	0.95 (1.82)	
		Alcohol use: 0–11	1.13 (1.67)	
		ADHD: 0–18	5.79 (4.29)	
		PTSD: 0–1	3.49 %	
NEET	Participants were categorized as NEET if they reported that they were not studying, working or undertaking vocational training at the time of the interview	–	11.57%	Goldman-Mellor *et al.* (2016)
Being a parent of an infant	Based on participants reporting either having given birth to or fathered a child	–	2.03%	–

s.d., Standard deviation; DSM-IV, Diagnostic and Statistical Manual of Mental Disorders, 4th edition; ADHD, attention-deficit/hyperactivity disorder; PTSD, post-traumatic stress disorder; NEET, not in employment, education or training.

#### Violence victimization

We assessed violence victimization between the ages of 12 and 18 years using the Juvenile Victimization Questionnaire (JVQ; Finkelhor *et al.* 2011). The JVQ contains questions covering seven forms of victimization: crime, peer/sibling, Internet, sexual, family, maltreatment and neglect. We asked participants to answer ‘yes’ or ‘no’ to each item, and when an instance of victimization was reported, further notes were taken about the details of the incident. Information from the JVQ was used to compile victimization ‘dossiers’ of each participant, which were coded for severity by four raters (Fisher *et al.* 2015). Overall severity of violence victimization was grouped into three categories: no exposure (47.6%), some exposure (28.1%) and severe exposure (24.3%).

We assessed exposure to maltreatment in childhood when participants were aged 5, 7, 10 and 12 years, via interviews with participants’ mothers. At age 5 years, assessments were based on the standardized clinical protocol from the MultiSite Child Development Project (Dodge *et al.* 1990; Lansford *et al.* 2002). At ages 7, 10 and 12 years this interview was modified to expand its coverage of contexts for child harm. Interviews were designed to enhance mothers’ comfort with reporting valid child maltreatment information, while also meeting researchers’ responsibilities for referral under the UK Children Act. We asked mothers whether either of their twins had been intentionally harmed (physically or sexually) by an adult or had contact with welfare agencies. Information on maltreatment collected over the years of data collection was compiled into a profile for each participant. These profiles were reviewed by two clinical psychologists and coded no harm (78.9%), probable harm (15.4%) and definite harm (5.7%).

### Data analysis

We used linear regressions to examine the associations between loneliness and overall sleep quality. To test for a specific profile of sleep complaints associated with loneliness, we conducted ordinal logistic regressions using each of the seven components of the PSQI. We verified the robustness of these associations first by controlling individually for each covariate (social isolation, depression, anxiety, alcohol use, ADHD, PTSD, NEET and being a parent of an infant), and finally by entering all covariates simultaneously in the model. As both loneliness and sleep quality were measured via self-report, we conducted a sensitivity analysis by testing the association between interviewer ratings of participants’ loneliness and self-reported sleep quality.

We controlled further for genetic and shared family factors using twin differences. MZ twin differences are attributable only to experiences unique to individuals, as the influence of genes and experiences within the family are held constant. Thus, if associations between loneliness and sleep quality are environmentally mediated, MZ twins who are lonelier than their co-twins would also have more sleep difficulties. To test this, we regressed the within-twin pair differences for sleep quality on the within-twin pair differences for loneliness. We conducted this analysis first on the whole sample, which controlled completely for the shared environment. We then repeated the analysis using MZ twins (*n* = 560 pairs), to control for both genetic and family environmental confounds.

We tested for an exacerbating effect of violence victimization on the association between loneliness and sleep quality using linear regression. In each analysis, we regressed sleep quality on loneliness, victimization and an interaction term (loneliness × victimization). We carried out this analysis first using adolescent victimization (age 12–18 years) as the moderator and, second, using childhood maltreatment (birth to age 12 years). As a further step, we repeated these analyses while controlling separately for covariates.

All analyses were conducted in Stata 14 (StataCorp, 2015). Participants in this study were pairs of same-sex twins, and therefore each family contained data for two individuals, resulting in non-independent observations. To correct for this, we used tests based on the Huber–White or sandwich variance (Williams, 2000), which adjusts the estimated standard errors to account for the dependence in the data.

## Results

### Associations between loneliness and sleep quality

Individuals who were lonelier reported worse overall sleep quality [*β* = 0.28, 95% confidence interval (CI) 0.24–0.33; Table 2]. Social isolation, depression, anxiety, alcohol use, ADHD, PTSD, NEET and being a parent of an infant were all associated with sleep quality over and above loneliness. However, none of these individual covariates explained the association between loneliness and sleep quality. When all covariates were entered simultaneously, this association reduced but remained significant (*β* = 0.07, 95% CI 0.02–0.12).

**Table 2.** Associations between loneliness and poor sleep quality in young adulthood, controlling for covariates^a^

	*β* (95% CI)								
	Baseline	Controlling for							
		Social isolation	Depression	Anxiety	Alcohol use	ADHD	PTSD	NEET	Being a parent
Loneliness	0.28 (0.24–0.33)	0.22 (0.17–0.27)	0.16 (0.12–0.21)	0.22 (0.17–0.26)	0.26 (0.22–0.31)	0.22 (0.17–0.26)	0.25 (0.20–0.29)	0.28 (0.23–0.32)	0.28 (0.23–0.33)
Covariate	–	0.15 (0.09–0.20)	0.30 (0.25–0.35)	0.19 (0.14–0.24)	0.20 (0.15–0.25)	0.23 (0.19–0.28)	0.16 (0.11–0.21)	0.07 (0.01–0.12)	0.05 (0.00–0.09)

*β*, Standardized regression coefficient; CI, confidence interval; ADHD, attention-deficit/hyperactivity disorder; PTSD, post-traumatic stress disorder; NEET, not in employment, education or training.

a All analyses adjusted for sex and socio-economic status.

Sensitivity analyses indicated that interviewer-rated loneliness was significantly associated with self-reported sleep quality, with a similar effect size to that of self-reported loneliness (*β* = 0.23, 95% CI 0.17–0.28). This association remained significant when controlling for each covariate. Entering social isolation into the model led to greater attenuation of the regression coefficient for interviewer-rated loneliness (43%) compared with that of self-reported loneliness (21%). Nonetheless, social isolation failed to fully account for the association.

Loneliness was significantly associated with each of the seven components of the PSQI (Table 3). However, after controlling for all covariates, loneliness remained independently associated specifically with poorer subjective sleep quality [odds ratio (OR) = 1.10, 95% CI 1.03–1.16] and greater daytime dysfunction (OR = 1.24, 95% CI 1.17–1.31).

**Table 3.** Associations between loneliness and components of sleep quality in young adulthood

	OR (95% CI)	
Component	Unadjusted^a^	Adjusted^b^
Subjective sleep quality	1.26 (1.20–1.32)*	1.09 (1.03–1.16)*
Sleep latency	1.19 (1.14–1.25)*	1.04 (0.99–1.10)
Sleep duration	1.15 (1.10–1.21)*	1.03 (0.97–1.09)
Habitual sleep efficiency	1.06 (1.01–1.11)*	0.96 (0.90–1.01)
Sleep disturbances	1.20 (1.14–1.27)*	1.03 (0.97–1.10)
Use of sleep medication	1.28 (1.15–1.42)*	0.94 (0.82–1.08)
Daytime dysfunction	1.43 (1.36–1.51)*	1.24 (1.17–1.31)*

OR, Odds ratio; CI, confidence interval; SES, socio-economic status; ADHD, attention-deficit/hyperactivity disorder; PTSD, post-traumatic stress disorder; NEET, not in employment, education or training.

a Adjusted for sex and SES.

b Adjusted for social isolation, depression, anxiety, alcohol use, ADHD, PTSD, NEET, being a parent, sex and SES.

* Significant association.

### Environmental association between loneliness and sleep quality

**Fig. 1.**
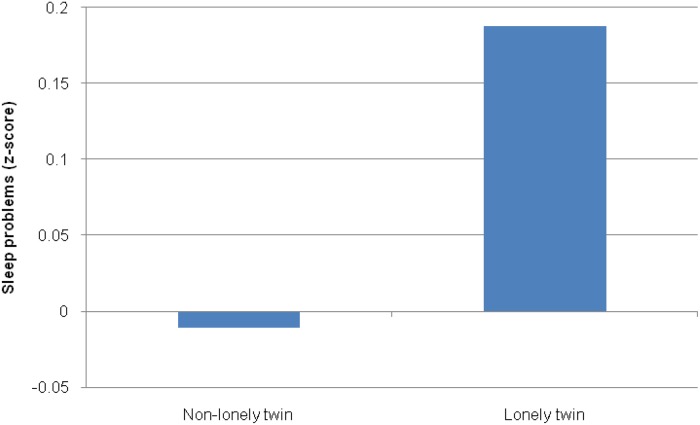
Mean (*z*-scored) sleep quality among 193 monozygotic twin pairs discordant for loneliness. Lonely and non-lonely groups were defined by taking a median split of the total loneliness score. For a colour figure, see the online version of the paper.

In the full sample (MZ and DZ twins), within-twin pair differences in loneliness were associated with within-pair differences in sleep quality (*β* = 0.20, 95% CI 0.14–0.27), indicating that the association between these experiences is independent of unmeasured influences from the family environment. Among MZ twins, within-pair differences in loneliness remained significantly associated with within-pair differences in sleep quality (*β* = 0.15, 95% CI 0.06–0.24). As shown in Fig. 1, among a subset of MZ pairs who were discordant for loneliness, the lonelier twins experienced poorer sleep quality than their non-lonely co-twins (Cohen's *d* = 0.20). This indicates an environmentally mediated association between loneliness and sleep which is independent of unmeasured genetic and family factors.

### Exacerbating effects of violence victimization on the association between loneliness and sleep quality

**Fig. 2.**
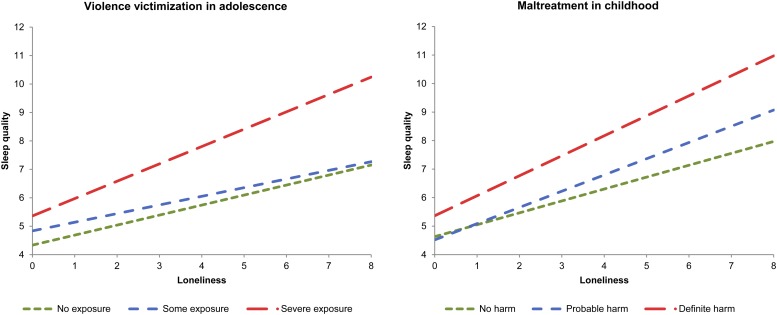
Exacerbating effect of violence victimization in adolescence and maltreatment in childhood on the association between loneliness and sleep quality in young adulthood. Higher scores on the *y*-axis reflect poorer sleep quality. For a colour figure, see the online version of the paper.

Individuals exposed to victimization during adolescence experienced both greater loneliness (*β* = 0.18, 95% CI 0.13–0.23) and worse sleep quality (*β* = 0.24, 95% CI 0.19–0.28) in early adulthood. Violence victimization moderated the association between loneliness and sleep quality, such that this association was stronger among those exposed to more severe victimization (interaction term: *β* = 0.06, 95% CI 0.02–0.10; Fig. 2). When controlling separately for depression and PTSD, the interaction term became non-significant, suggesting that symptoms of psychopathology may be a pathway through which adolescent victimization moderates the association between loneliness and sleep quality.

When looking at the effect of victimization in childhood, individuals who experienced definite harm were lonelier (*β* = 0.07, 95% CI 0.02–0.12) and had poorer sleep quality (*β* = 0.10, 95% CI 0.05–0.16) in early adulthood. Childhood maltreatment showed a similar moderating effect to that of adolescent victimization; the association between loneliness and sleep was exacerbated among those exposed to maltreatment (interaction term: *β* = 0.05, 95% CI 0.01–0.09; Fig. 2). This effect was not explained by controls for covariates.

### Sex differences

Loneliness did not differ by sex. Females had on average poorer sleep quality than males (mean = 5.69 *v*. 5.07, respectively; *p* < 0.001). No sex differences were detected in the associations between loneliness and sleep quality.

## Discussion

In line with previous studies (Cacioppo *et al.* 2002; Hawkley *et al.* 2010*a*; Kurina *et al.* 2011), we showed a modest but robust association between loneliness and poorer sleep quality in a nationally representative cohort of young adults. We furthered this evidence by showing that the association was not explained by loneliness and sleep quality having common genetic origins or by confounding influences from the environments shared by twins. Further, the strength of this association in young adulthood was moderated by exposure to violence during childhood and adolescence. In the case of more recent exposure in adolescence, symptoms of depression or PTSD may be pathways through which victimization exerts this exacerbating effect.

Although the associations between loneliness and sleep quality were small, they emerged from a thorough and stringent test for an independent association, controlling for many other factors which may explain their co-occurrence. In particular, the MZ twin differences method is a robust test which controls comprehensively for unobserved sources of variation within families and other environments shared by siblings. Furthermore, loneliness is often accompanied by depressive symptoms (Cacioppo *et al.* 2006*b*; Matthews *et al.* 2016), which in turn have their own negative effects on sleep. Of all the covariates investigated, depression was the most strongly associated with sleep problems; a finding that is not unexpected given that changes in sleep are included among the diagnostic criteria for major depression (American Psychiatric Association, 2013). However, the association between loneliness and sleep problems was independent of the contribution of depression.

Impoverished social connections increase the risk of numerous negative health outcomes, including elevated blood pressure (Hawkley *et al.* 2010*b*), impaired immune functioning (Pressman *et al.* 2005), obesity (Lauder *et al.* 2006) and mortality (Holt-Lunstad *et al.* 2015). Deficiencies in the quantity and quality of sleep are predictive of similar health problems (Irwin, 2002; Gangwisch *et al.* 2006; Patel & Hu, 2008; Cappucio *et al.* 2010). Markers of long-term health outcomes are unlikely to be detectable at the young age of the present cohort. Nonetheless, our findings indicate that a relationship between loneliness and reduced sleep quality is already present in young adulthood. Longitudinal research is necessary to test whether the foreshortened lifespan observed in chronically lonely individuals is explained, in part, by the effects of diminished sleep quality. Furthermore, while young adulthood is an important period for the formation of social relationships, loneliness could also have roots in early attachment problems; therefore, a further goal for future research should be to investigate the potential role of attachment styles in explaining or modifying the relationship between loneliness and sleep.

We identified a subgroup of lonely individuals exposed to violence victimization who were particularly vulnerable to experiencing poorer sleep quality. Loneliness is associated with changes in cognition that include raised vigilance for threats in the environment (Cacioppo *et al.* 2006*a*; Cacioppo & Hawkley, 2009). Past exposure to actual threats such as victimization may establish a pre-existing vulnerability for loneliness to act upon, by reinforcing perceptions of others’ intent to harm. To the extent that vigilance for threats undermines the restfulness of sleep, this may account for the exacerbating effect of violence victimization that we detected. It should be noted, however, that there could be other psychological processes that mediate the relationship between loneliness and sleep problems, such as rumination. Further investigation is warranted to investigate the range of pathways through which loneliness may intrude on sleep.

Some physiological processes may also explain the association between loneliness and sleep quality. A first possible candidate is the stress response. Loneliness is associated with changes in circulating cortisol, indicating elevated hypothalamic–pituitary–adrenal axis activation (Pressman *et al.* 2005; Doane & Adam, 2010). Physiological arousal resulting from this process may play a role in the disrupted sleep of lonely individuals. Second, when looking at individual items, lonely individuals reported two out of 10 specific sources of sleep disturbance: feeling too cold, and having bad dreams. Whilst this finding should be interpreted with caution, it suggests potential avenues of further investigation. For instance, experimental research has found an association between social exclusion and reductions in perceived ambient temperature (Zhong & Leonardelli, 2008). Further, dream disturbances are associated with greater stress and anxiety (Levin & Nielsen, 2007), and may represent a further manifestation of emotional distress in lonely individuals.

Our study has some limitations. Loneliness and sleep quality were measured cross-sectionally, and no conclusions can be drawn about the directionality of the associations. Daytime dysfunction may, for example, contribute to stability or increases in loneliness by hindering social interactions. However, prior longitudinal findings indicate that loneliness more strongly predicts subsequent sleep quality, rather than vice-versa (Hawkley *et al.* 2010*a*). Second, by virtue of the twin design, all participants had at least one sibling, which should reduce loneliness on average. Twins are same-age siblings who may feel closer than typical siblings. If this is true, then rates of loneliness and effects of loneliness may be underestimated in our sample.

A third limitation is that both loneliness and sleep quality were measured via self-report. However, further analyses showed that the associations between loneliness and sleep quality were replicated when interviewers’ ratings were substituted for self-reported loneliness. Interestingly, controlling for social isolation led to greater attenuation of the association between loneliness and sleep quality when loneliness was measured via interviewer report. A potential explanation for this is that independent observers are more likely to conflate social isolation and loneliness in others, in which case using self-reports is a strength, rather than a weakness, when assessing loneliness. It remains possible, however, that self-reports of sleep quality may be vulnerable to reporting bias. Polysomnography and actigraphy measure aspects of sleep more objectively but are less practical to implement in large cohort studies involving comprehensive interview assessments. Nonetheless, the PSQI is a well-validated and widely used instrument, and is correlated with other measurement approaches such as sleep diaries (Buysse *et al.* 1989; Backhaus *et al.* 2002). Different methods each provide unique information about sleep, and there is value in using a variety of approaches to build a thorough profile of sleep impairments (Gregory & Sadeh, 2016). Our findings complement those of studies using objective measures (Cacioppo *et al.* 2002; Kurina *et al.* 2011), showing that lonely individuals experience more fragmented sleep.

## Conclusions

Diminished sleep quality is one of the many ways in which loneliness gets ‘under the skin’, and our findings underscore the importance of early intervention to reduce loneliness in young people, which may be the starting point for a cascade of physical health problems in later life. Studies of interventions to reduce loneliness suggest that resolving negative social cognitions that can damage social interactions is an expedient strategy (Masi *et al.* 2011; Cacioppo *et al.* 2015). Further, our findings suggest that interventions should consider not only individuals’ current social circumstances, but also influences of past experiences including violence victimization, which may constitute a pre-existing vulnerability that exacerbates the effects of loneliness. Future research should explore in more detail loneliness's relationship with victimization and other forms of trauma.
